# Supplementary material for: A Likelihood Approach for Real-Time Calibration of Stochastic Compartmental Epidemic Models
Source: PLoS Comput Biol. 2017 Jan 17;13(1):e1005257. doi: 10.1371/journal.pcbi.1005257 (PMC5240920; doi:10.1371/journal.pcbi.1005257)
Supplement: S1 File — (TAR.GZ) [file pcbi.1005257.s014.tar.gz › HSPH_Online-SI-Revision/IPoi09/n100-severe/n100-severe_BENCH2_table-8.pdf]

1	newly 3 weeks spec	2276.	3773.47	{1169., 5252.}	83.4567	0.282026	newly 3 weeks cumul	4764.	9697.21	{1990., 14838.}	122.863	0.35896	newly 5 weeks spec	4385.	3789.89	{2365., 4712.}	17.6211	0.0968611	newly 5 weeks cumul	12438.	17466.9	{6127., 23432.}	58.2286	0.221156	newly 58 weeks cumul	64638.	33462.7	{30045., 38583.}	48.2306	0.288778
2	newly 3 weeks spec	951.	4529.39	{1026., 9112.}	383.04	0.595057	newly 3 weeks cumul	1661.	6707.1	{1367., 14084.}	314.212	0.537171	newly 5 weeks spec	4438.	14581.1	{8816., 19031.}	229.359	0.494376	newly 5 weeks cumul	8385.	30917.5	{13611., 47221.}	271.069	0.528137	newly 65 weeks cumul	54065.	80467.8	{56883., 93014.}	48.8353	0.167477
3	newly 3 weeks spec	788.	3869.43	{0., 9510.}	416.266	0.979453	newly 3 weeks cumul	1184.	5446.43	{53., 12711.}	386.578	0.845189	newly 5 weeks spec	4947.	13852.7	{0., 21443.}	201.762	0.927738	newly 5 weeks cumul	8233.	28333.3	{53., 51696.}	265.849	0.87501	newly 66 weeks cumul	68234.	69489.1	{53., 93075.}	32.4609	0.588696
4	newly 3 weeks spec	1410.	10593.6	{2369., 16788.}	653.148	0.808115	newly 3 weeks cumul	2503.	18965.2	{3476., 34512.}	660.574	0.795518	newly 5 weeks spec	4326.	14288.	{10245., 17411.}	230.283	0.508628	newly 5 weeks cumul	9460.	47343.4	{22419., 64012.}	400.764	0.671101	newly 64 weeks cumul	66493.	81424.1	{62674., 92431.}	24.1297	0.0920102
5	newly 3 weeks spec	2748.	4793.61	{1490., 6567.}	87.9852	0.290676	newly 3 weeks cumul	5863.	13293.6	{2699., 18765.}	141.343	0.396781	newly 5 weeks spec	4410.	4153.56	{1712., 6288.}	33.3107	0.171957	newly 5 weeks cumul	13943.	22090.7	{9013., 27779.}	69.8204	0.242985	newly 58 weeks cumul	66068.	35197.	{22181., 42779.}	46.7261	0.283708
6	newly 3 weeks spec	1016.	7776.56	{3191., 10727.}	672.51	0.890778	newly 3 weeks cumul	1815.	16809.	{5727., 25146.}	833.217	0.949695	newly 5 weeks spec	3575.	7588.26	{3593., 10181.}	120.76	0.360701	newly 5 weeks cumul	7380.	32872.8	{19963., 42813.}	352.438	0.677584	newly 64 weeks cumul	66876.	47913.1	{41776., 56429.}	28.3552	0.14684
7	newly 3 weeks spec	815.	2088.72	{128., 5310.}	186.004	0.463074	newly 3 weeks cumul	1311.	3125.26	{247., 8493.}	171.032	0.444169	newly 5 weeks spec	3565.	8961.62	{1620., 14747.}	172.652	0.461201	newly 5 weeks cumul	6614.	16945.6	{2299., 31203.}	179.112	0.463585	newly 65 weeks cumul	69295.	81097.8	{60823., 93484.}	23.7846	0.0947764
8	newly 3 weeks spec	1680.	4688.53	{2375., 6023.}	179.079	0.424023	newly 3 weeks cumul	3165.	11497.6	{4434., 16681.}	263.273	0.521297	newly 5 weeks spec	3946.	4562.47	{2707., 5361.}	26.5062	0.107214	newly 5 weeks cumul	9677.	20963.9	{13477., 24997.}	117.304	0.324461	newly 61 weeks cumul	59021.	32801.9	{28173., 35628.}	44.4234	0.256546
9	newly 3 weeks spec	240.	195.917	{0., 561.}	90.4051	1.01772	newly 3 weeks cumul	369.	329.361	{0., 996.}	94.6176	0.986967	newly 5 weeks spec	1645.	834.528	{0., 2106.}	78.7622	1.24026	newly 5 weeks cumul	2651.	1562.39	{0., 3847.}	78.2472	1.17921	newly 66 weeks cumul	62553.	52013.8	{0., 90576.}	46.6827	1.08927
10	newly 3 weeks spec	1249.	1590.28	{235., 3484.}	72.409	0.35266	newly 3 weeks cumul	2193.	3608.39	{420., 8369.}	102.936	0.385083	newly 5 weeks spec	4136.	2442.83	{568., 3821.}	40.9373	0.354802	newly 5 weeks cumul	8808.	8046.33	{1385., 15841.}	49.7843	0.336738	newly 59 weeks cumul	62587.	36300.3	{32706., 38079.}	42.0003	0.237106
11	newly 3 weeks spec	804.	4756.79	{1474., 8444.}	498.916	0.826386	newly 3 weeks cumul	1294.	8587.64	{2481., 17719.}	571.384	0.865097	newly 5 weeks spec	3948.	7364.	{5109., 9319.}	93.8721	0.432513	newly 5 weeks cumul	7140.	22571.3	{13236., 31808.}	223.687	0.648263	newly 65 weeks cumul	63055.	37976.1	{37356., 42337.}	39.773	0.404654
12	newly 3 weeks spec	2111.	3980.79	{1237., 5794.}	105.41	0.345665	newly 3 weeks cumul	4254.	9832.89	{2675., 14429.}	147.657	0.417799	newly 5 weeks spec	4443.	4402.75	{2421., 5910.}	26.0128	0.141215	newly 5 weeks cumul	11913.	18607.8	{6968., 25343.}	72.9152	0.273854	newly 58 weeks cumul	66884.	37813.4	{28465., 46802.}	43.4643	0.254621
13	newly 3 weeks spec	2345.	4793.61	{1490., 6567.}	116.472	0.344245	newly 3 weeks cumul	5116.	13293.6	{2699., 18765.}	173.337	0.442818	newly 5 weeks spec	4572.	4153.56	{1712., 6288.}	32.1304	0.171957	newly 5 weeks cumul	13208.	22090.7	{9013., 27779.}	78.0339	0.261278	newly 58 weeks cumul	66392.	35197.	{22181., 42779.}	46.9861	0.285833
14	newly 3 weeks spec	1956.	3979.79	{1772., 5100.}	110.193	0.316506	newly 3 weeks cumul	4187.	11039.2	{3471., 15135.}	171.591	0.415197	newly 5 weeks spec	3466.	3440.95	{2131., 4957.}	23.9682	0.10779	newly 5 weeks cumul	10276.	18270.1	{8998., 22194.}	84.2612	0.263487	newly 58 weeks cumul	61739.	30824.9	{25765., 38292.}	50.0722	0.308063
15	newly 3 weeks spec	1396.	3195.24	{1089., 4786.}	143.212	0.39404	newly 3 weeks cumul	2586.	7610.86	{2212., 12916.}	207.13	0.473255	newly 5 weeks spec	3233.	3877.95	{2341., 4956.}	35.4028	0.164748	newly 5 weeks cumul	7869.	15156.5	{6155., 21455.}	106.738	0.334457	newly 60 weeks cumul	52704.	34123.7	{30452., 36683.}	35.2541	0.189846
16	newly 3 weeks spec	1498.	4456.39	{1983., 6590.}	208.908	0.479147	newly 3 weeks cumul	2968.	10311.6	{3841., 16698.}	259.164	0.530269	newly 5 weeks spec	3246.	5706.09	{4144., 6895.}	85.2474	0.276423	newly 5 weeks cumul	8486.	21303.4	{11770., 29122.}	161.491	0.414997	newly 59 weeks cumul	63449.	48571.4	{43935., 51565.}	23.4481	0.117193
17	newly 3 weeks spec	711.	5178.9	{789., 9250.}	633.004	0.785709	newly 3 weeks cumul	1220.	9275.24	{1187., 17719.}	666.422	0.795616	newly 5 weeks spec	2605.	7884.97	{3923., 11946.}	207.107	0.475365	newly 5 weeks cumul	5099.	24428.9	{6884., 34395.}	383.773	0.656253	newly 65 weeks cumul	53195.	42465.5	{37712., 56171.}	22.0913	0.110479
18	newly 3 weeks spec	1434.	6415.36	{926., 10359.}	362.384	0.753558	newly 3 weeks cumul	2751.	12363.8	{1559., 22346.}	365.335	0.754879	newly 5 weeks spec	4058.	8846.08	{3769., 10816.}	129.226	0.480683	newly 5 weeks cumul	9240.	29661.4	{7314., 40218.}	234.276	0.650348	newly 63 weeks cumul	55677.	52097.9	{49635., 55877.}	9.62895	0.189415
19	newly 3 weeks spec	1846.	3565.16	{843., 7000.}	116.767	0.331108	newly 3 weeks cumul	3207.	6086.54	{1308., 12497.}	117.319	0.331518	newly 5 weeks spec	5657.	9595.36	{4432., 14183.}	80.0759	0.256735	newly 5 weeks cumul	12216.	22071.5	{7676., 37647.}	95.7157	0.287968	newly 63 weeks cumul	62777.	79649.6	{57018., 91260.}	29.7479	0.111352
20	newly 3 weeks spec	1317.	1418.92	{178., 2659.}	65.2081	0.354919	newly 3 weeks cumul	2412.	2563.1	{330., 4805.}	66.2521	0.359633	newly 5 weeks spec	4511.	4108.18	{725., 7149.}	46.9708	0.297457	newly 5 weeks cumul	9394.	9196.89	{1418., 16639.}	54.876	0.322461	newly 61 weeks cumul	61521.	87882.1	{83660., 93231.}	42.8489	0.1538
21	newly 3 weeks spec	780.	6416.25	{1976., 10468.}	727.532	0.918127	newly 3 weeks cumul	1243.	10356.7	{2859., 16455.}	738.129	0.886368	newly 5 weeks spec	3321.	14392.6	{9483., 17824.}	337.229	0.705429	newly 5 weeks cumul	6292.	36029.3	{17387., 48350.}	476.464	0.78873	newly 65 weeks cumul	63478.	76239.9	{61356., 92192.}	26.8653	0.177438
22	newly 3 weeks spec	720.	3726.23	{181., 6014.}	446.688	0.833528	newly 3 weeks cumul	1304.	8350.88	{288., 15192.}	569.476	0.862953	newly 5 weeks spec	2369.	4662.58	{741., 8537.}	123.946	0.563452	newly 5 weeks cumul	4863.	17413.5	{1401., 28322.}	285.655	0.694545	newly 63 weeks cumul	55662.	36600.3	{26648., 53850.}	35.8004	0.297163
23	newly 3 weeks spec	325.	1973.74	{0., 4007.}	557.575	1.22929	newly 3 weeks cumul	484.	2533.26	{0., 5403.}	475.377	1.16836	newly 5 weeks spec	3196.	11838.3	{0., 24230.}	317.607	1.37052	newly 5 weeks cumul	4636.	20443.1	{0., 46966.}	388.235	1.37281	newly 67 weeks cumul	66380.	65221.1	{0., 92940.}	42.7683	1.14648
24	newly 3 weeks spec	356.	3207.14	{0., 10728.}	919.506	2.1837	newly 3 weeks cumul	524.	3739.71	{0., 12511.}	733.029	2.13762	newly 5 weeks spec	2825.	8071.13	{0., 27675.}	302.143	2.58901	newly 5 weeks cumul	4283.	18679.9	{0., 66809.}	452.444	2.61897	newly 68 weeks cumul	68010.	43831.9	{0., 92518.}	62.1823	2.73134
25	newly 3 weeks spec	331.	354.96	{0., 250.}	179.353	2.44801	newly 3 weeks cumul	441.	414.95	{0., 351.}	165.449	2.28357	newly 5 weeks spec	2373.	2329.15	{0., 6836.}	167.137	3.00692	newly 5 weeks cumul	3717.	4180.74	{0., 9055.}	181.911	2.83809	newly 68 weeks cumul	58819.	37389.1	{0., 91225.}	73.6216	2.91321
26	newly 3 weeks spec	1190.	638.803	{59., 1432.}	63.3324	0.563885	newly 3 weeks cumul	2010.	1176.77	{99., 2689.}	64.6742	0.551494	newly 5 weeks spec	4173.	1940.51	{235., 4026.}	58.0076	0.542711	newly 5 weeks cumul	8492.	4250.57	{419., 8826.}	59.9142	0.548296	newly 62 weeks cumul	69666.	83628.1	{74230., 93128.}	24.2025	0.0959873
27	newly 3 weeks spec	974.	743.905	{182., 1509.}	56.3606	0.393271	newly 3 weeks cumul	1673.	1272.68	{296., 2605.}	57.6723	0.398704	newly 5 weeks spec	3834.	2788.6	{844., 5298.}	47.6505	0.349968	newly 5 weeks cumul	7556.	5556.98	{1633., 10621.}	51.0609	0.367545	newly 63 weeks cumul	62217.	86364.4	{71187., 93332.}	38.8116	0.14072
28	newly 3 weeks spec	2141.	8525.35	{4354., 10696.}	306.216	0.623855	newly 3 weeks cumul	4227.	19834.4	{7472., 27456.}	377.417	0.678458	newly 5 weeks spec	4410.	7040.04	{4486., 9982.}	67.3834	0.2272	newly 5 weeks cumul	11858.	35363.4	{24158., 41530.}	205.867	0.505841	newly 62 weeks cumul	54529.	49621.6	{45638., 53405.}	8.99968	0.0417496
29	newly 3 weeks spec	1545.	4844.86	{1563., 8328.}	219.79	0.444597	newly 3 weeks cumul	2666.	9685.62	{2759., 18935.}	270.103	0.487352	newly 5 weeks spec	5090.	7212.81	{5118., 8373.}	46.5348	0.164405	newly 5 weeks cumul	10609.	23304.8	{10795., 34983.}	125.104	0.327046	newly 63 weeks cumul	65892.	47548.6	{44256., 51696.}	27.8386	0.142602
30	newly 3 weeks spec	1626.	2077.78	{354., 4178.}	75.9491	0.351819	newly 3 weeks cumul	3106.	4091.53	{685., 8457.}	80.8364	0.361836	newly 5 weeks spec	3975.	4593.07	{1064., 8012.}	56.4417	0.283509	newly 5 weeks cumul	9727.	11900.5	{2388., 22731.}	67.0421	0.31893	newly 59 weeks cumul	54604.	80420.5	{73736., 88005.}	47.2794	0.16704
31	newly 3 weeks spec	312.	188.2	{0., 179.}	133.897	2.33092	newly 3 weeks cumul	454.	228.88	{0., 237.}	123.555	2.1665	newly 5 weeks spec	3043.	1879.18	{0., 2344.}	134.978	3.01755	newly 5 weeks cumul	4393.	2932.35	{0., 3251.}	139.776	2.76503	newly 68 weeks cumul	60937.	40862.6	{0., 92226.}	71.8851	2.72128
32	newly 3 weeks spec	434.	543.939	{49., 1110.}	85.8749	0.464672	newly 3 weeks cumul	710.	794.106	{81., 1592.}	77.0102	0.465657	newly 5 weeks spec	2775.	3722.95	{325., 7635.}	83.4502	0.468658	newly 5 weeks cumul	4597.	6036.02	{508., 12468.}	83.7585	0.478269	newly 66 weeks cumul	62201.	84156.8	{73785., 93680.}	43.0135	0.320556
33	newly 3 weeks spec	975.	5188.17	{916., 10906.}	442.442	0.724968	newly 3 weeks cumul	1516.	7047.34	{1225., 15502.}	376.731	0.673128	newly 5 weeks spec	5236.	18874.3	{10441., 22687.}	266.84	0.636867	newly 5 weeks cumul	8992.	38609.5	{14418., 56507.}	335.868	0.698178	newly 66 weeks cumul	68723.	84748.6	{76073., 93822.}	26.9219	0.195325
34	newly 3 weeks spec	1779.	1371.05	{373., 3118.}	57.3197	0.373012	newly 3 weeks cumul	3127.	2786.83	{692., 6736.}	62.0893	0.360294	newly 5 weeks spec	4645.	2973.47	{992., 5683.}	47.1862	0.342512	newly 5 weeks cumul	10816.	7837.12	{2325., 15832.}	52.6885	0.354807	newly 58 weeks cumul	64121.	79504.1	{69372., 87413.}	27.6411	0.107102
35	newly 3 weeks spec	295.	163.323	{2., 336.}	95.7135	1.11657	newly 3 weeks cumul	455.	237.935	{7., 513.}	91.7476	0.986896	newly 5 weeks spec	2408.	1235.34	{5., 2832.}	95.8331	1.36458	newly 5 weeks cumul	3628.	1975.6	{25., 4218.}	96.7924	1.20414	newly 67 weeks cumul	61775.	73203.5	{165., 93684.}	43.6174	0.613361
36	newly 3 weeks spec	489.	219.532	{31., 474.}	63.6091	0.581761	newly 3 weeks cumul	783.	358.968	{64., 741.}	61.9948	0.553634	newly 5 weeks spec	2506.	1069.44	{124., 2252.}	63.2167	0.610533	newly 5 weeks cumul	4350.	1925.55	{213., 4039.}	62.7119	0.580709	newly 65 weeks cumul	66421.	87130.3	{79698., 93450.}	31.9558	0.120022
37	newly 3 weeks spec	391.	8185.48	{0., 16864.}	2030.51	1.74693	newly 3 weeks cumul	633.	12220.5	{0., 26687.}	1867.6	1.74016	newly 5 weeks spec	3280.	7745.19	{0., 15891.}	174.322	1.17904	newly 5 weeks cumul	5202.	30450.8	{0., 45780.}	522.404	1.56569	newly 67 weeks cumul	56370.	35917.8	{0., 48238.}	36.2821	1.15288
38	newly 3 weeks spec	728.	1302.51	{158., 2970.}	125.	0.472099	newly 3 weeks cumul	1154.	1992.84	{229., 4805.}	120.402	0.441264	newly 5 weeks spec	4350.	6248.75	{1109., 12175.}	84.987	0.390663	newly 5 weeks cumul	7337.	11362.3	{1757., 23536.}	97.8738	0.411765	newly 65 weeks cumul	69743.	83332.3	{70650., 93466.}	26.575	0.150885
39	newly 3 weeks spec	1971.	9152.15	{5517., 10781.}	364.341	0.655024	newly 3 weeks cumul	3791.	23893.2	{9912., 30107.}	530.261	0.771863	newly 5 weeks spec	4327.	6323.46	{3277., 10005.}	56.0168	0.183744	newly 5 weeks cumul	11151.	38436.7	{29678., 42298.}	244.693	0.533084	newly 62 weeks cumul	53564.	49203.8	{41607., 55696.}	9.44995	0.0447302
40	newly 3 weeks spec	1025.	10302.2	{2570., 16982.}	910.101	0.943722	newly 3 weeks cumul	1775.	18346.	{3839., 30987.}	939.167	0.942713	newly 5 weeks spec	3528.	10232.3	{7275., 13516.}	190.032	0.445279	newly 5 weeks cumul	7401.	40707.6	{23087., 56653.}	451.742	0.716966	newly 65 weeks cumul	51673.	55434.5	{52307., 63007.}	7.27936	0.0282149
41	newly 3 weeks spec	676.	17707.5	{2071., 31030.}	2537.	1.61146	newly 3 weeks cumul	1025.	22861.9	{2402., 42324.}	2147.97	1.55182	newly 5 weeks spec	4452.	18297.8	{5895., 28423.}	328.547	0.986004	newly 5 weeks cumul	7242.	67410.6	{41943., 83772.}	848.373	1.34287	newly 67 weeks cumul	64974.	81072.5	{74752., 93008.}	42.3207	0.633329
42	newly 3 weeks spec	1593.	2910.65	{398., 5735.}	115.304	0.388255	newly 3 weeks cumul	2901.	6029.28	{675., 12557.}	139.773	0.412636	newly 5 weeks spec	4138.	5216.15	{1382., 7513.}	56.0331	0.262757	newly 5 weeks cumul	9608.	15322.2	{2819., 27551.}	90.37	0.338664	newly 60 weeks cumul	61993.	60140.2	{51192., 75171.}	13.7493	0.0606207
43	newly 3 weeks spec	1692.	3771.88	{747., 6695.}	143.315	0.387619	newly 3 weeks cumul	3552.	8672.04	{1303., 13847.}	166.954	0.426203	newly 5 weeks spec	3812.	4986.	{2075., 8768.}	49.4269	0.185141	newly 5 weeks cumul	9984.	18185.2	{4895., 31148.}	101.804	0.314829	newly 59 weeks cumul	48969.	44573.3	{32842., 66523.}	29.3684	0.136497
44	newly 3 weeks spec	459.	693.137	{83., 1488.}	110.859	0.561295	newly 3 weeks cumul	732.	1010.16	{167., 2066.}	101.2	0.505714	newly 5 weeks spec	2554.	4478.96	{590., 8922.}	122.17	0.602687	newly 5 weeks cumul	4392.	7385.69	{934., 15979.}	119.097	0.558356	newly 66 weeks cumul	67035.	79707.8	{56298., 93532.}	33.6579	0.389867
45	newly 3 weeks spec	956.	5481.78	{1607., 9356.}	481.867	0.738146	newly 3 weeks cumul	1721.	10426.8	{2985., 18259.}	514.309	0.73686	newly 5 weeks spec	3513.	7975.35	{5393., 10292.}	135.484	0.417874	newly 5 weeks cumul	7079.	25751.3	{11855., 35997.}	272.239	0.594584	newly 64 weeks cumul	64288.	48281.6	{45856., 49149.}	24.8979	0.124994
46	newly 3 weeks spec	720.	855.333	{98., 1914.}	89.2003	0.482132	newly 3 weeks cumul	1225.	1696.45	{284., 3981.}	101.363	0.440342	newly 5 weeks spec	3211.	2073.45	{301., 3950.}	55.3165	0.540565	newly 5 weeks cumul	5975.	5140.7	{595., 10751.}	64.3865	0.457982	newly 63 weeks cumul	66403.	51488.9	{34024., 90547.}	35.8971	0.282308
47	newly 3 weeks spec	2512.	4858.24	{1250., 6251.}	109.404	0.355623	newly 3 weeks cumul	5499.	12848.3	{2221., 19461.}	151.101	0.430679	newly 5 weeks spec	4726.	4426.65	{2160., 6342.}	31.407	0.162275	newly 5 weeks cumul	13753.	22249.9	{8376., 26607.}	75.7905	0.27243	newly 59 weeks cumul	63771.	35867.4	{27496., 41228.}	43.7559	0.254067
48	newly 3 weeks spec	1312.	5975.88	{656., 11160.}	371.803	0.613581	newly 3 weeks cumul	2348.	10178.3	{987., 20366.}	353.473	0.597003	newly 5 weeks spec	4095.	11432.	{4136., 14312.}	180.801	0.420728	newly 5 weeks cumul	8888.	31086.7	{6708., 46819.}	257.291	0.514521	newly 64 weeks cumul	66105.	65214.2	{60814., 78440.}	9.28266	0.0394064
49	newly 3 weeks spec	197.	1722.99	{0., 3990.}	907.061	2.07667	newly 3 weeks cumul	276.	2003.82	{0., 4405.}	756.891	1.98929	newly 5 weeks spec	2370.	5430.92	{0., 25921.}	264.32	2.58658	newly 5 weeks cumul	3259.	11804.2	{0., 57519.}	396.448	2.53064	newly 68 weeks cumul	66569.	43942.9	{0., 92455.}	63.3931	2.65005
50	newly 3 weeks spec	580.	9075.94	{0., 19277.}	1492.51	1.499	newly 3 weeks cumul	912.	11582.6	{0., 24201.}	1198.28	1.44094	newly 5 weeks spec	4132.	19621.7	{0., 29365.}	402.564	1.26442	newly 5 weeks cumul	6745.	50286.3	{0., 74373.}	673.226	1.45887	newly 67 weeks cumul	62718.	71190.1	{0., 92674.}	43.7749	0.913268
